# Alkylation of Methyl Linoleate with Propene in Ionic Liquids in the Presence of Metal Salts

**DOI:** 10.3390/molecules201219805

**Published:** 2015-12-07

**Authors:** Christian Silvio Pomelli, Tiziana Ghilardi, Cinzia Chiappe, Alberto Renato de Angelis, Vincenzo Calemma

**Affiliations:** 1 Dipartimento di Farmacia, Università di Pisa, via Bonanno 33, Pisa 56126, Italy; christian.pomelli@unipi.it (C.S.P.); tiz.ghilardi@gmail.com (T.G.); 2 ENI Downstream R & D, Development Operations and Technology, S. Donato Milanese (Mi) 20097, Italy; vincenzo.calemma@eni.com

**Keywords:** ionic liquid, metal salt, methyl linoleate alkylation

## Abstract

Vegetable oils and fatty acid esters are suitable precursor molecules for the production of a variety of bio-based products and materials, such as paints and coatings, plastics, soaps, lubricants, cosmetics, pharmaceuticals, printing inks, surfactants, and biofuels. Here, we report the possibility of using Lewis acidic ionic liquids (ILs) to obtain polyunsaturated ester dimerization-oligomerization and/or, in the presence of another terminal alkene (propene), co-polymerization. In particular, we have tested the Lewis acidic mixtures arising from the addition of a proper amount of GaCl_3_ (Χ > 0.5) to two chloride-based (1-butyl-3-methylimidazolium chloride, [bmim]Cl, and 1-butylisoquinolium chloride, [BuIsoq]Cl) or by dissolution of a smaller amount of Al(Tf_2_N)_3_ (Χ = 0.1) in 1-butyl-3-methylimidazolium bis(trifluoromethylsulfonyl)imide, [bmim][Tf_2_N]. On the basis of product distribution studies, [bmim][Tf_2_N]/Al(Tf_2_N)_3_ appears the most suitable medium in which methyl linoleate alkylation with propene can compete with methyl linoleate or propene oligomerization.

## 1. Introduction

Biomass feedstocks can be classified into three basic categories: lignocellulosics, amorphous sugars, and triglycerides, including vegetable oils and animal fats. With few exceptions, vegetable oils consist almost entirely of triacylglycerol esters based on fatty acids with chain lengths of C8–C24, with C16 and C18 being the most common. Plant oils are used primarily for food (81.5%) and feed (5.2%) purposes, although oils are increasingly being utilized as renewable sources of industrial feedstocks and fuel (14.2%) [1]. Vegetable oils are indeed suitable precursor molecules for the production of a variety of bio-based products and materials, such as paints and coatings, plastics, soaps, lubricants, cosmetics, pharmaceuticals, printing inks, surfactants, and biofuels [2]. The demand for vegetable oils has increased rapidly in the past decade, as a result of a combination of social and economic factors, and is expected to increase in the near future.

Constituted by fatty acids with alkyl chains of different length, degree of unsaturation, position, and geometry of the double bond(s), vegetable oils present different physico-chemical properties that largely affect their chemical usage. For example, oils containing high concentrations of saturated fatty acids are mainly used in soaps and surfactants while polyunsaturated oils (containing mainly linoleic (C18:2) and linolenic (C18:3) acids) are used as drying agents for paints and coatings [3,4]. Castor oil, with a high content of ricinoleic acid (12-OH-9-cis C18:1), is an attractive renewable feedstock for oligo- and polymerization processes [5,6].

Today, the chemical and health-related industries prefer plant oils to fossil oils only when the former contain high-value fatty acids [2] and the processing costs to obtain the final products are low [7]. However, plant oils could provide renewable and environmentally friendly replacements for many other fossil-based raw materials, in particular if efficient processes to obtain the desired products are developed and if the availability of vegetable oils meets industrial demands. 

Among the possible applications, the use of oils as renewable materials in the active field of lubrication is increasing rapidly due to their biodegradability, low ecotoxicity, and excellent tribological properties [8]. Bio-based lubricants generally have lower coefficients of friction, improved wear characteristics, higher viscosity indexes, and lower volatility and flashpoint than mineral-based oils [9,10]. However, they suffer from poor low-temperature properties and oxidative stability [8]. These problems can be overcome by using additives and synthetic fluids [8], whereas their transformation in higher-molecular-weight products by thermal polymerization [11] can provide fluids with sufficient viscosity for gear oil applications. Vegetable oil polymerization is indeed a procedure often carried out on polyunsaturated oils and it is also exploited for other industrial applications, for example to obtain inks, surfactants, and composites [12,13]. 

It is well known that polyunsaturated oils contain generally non-conjugated *cis* double bonds. However, conjugated vegetable oils, characterized by a significantly higher reactivity in many processes involving double bonds, including polymerization, can be synthesized from vegetable oils rich in linoleic acid using different catalytic processes (under basic or acid conditions) or by photoisomerization [14]. The classic alkaline process produces almost equimolar mixtures of c9,t11 and t10,c12 isomers but, despite the high product yield [15,16], it has several ecological drawbacks. A recent review discusses the state-of-the-art of catalytic processes to transform vegetable oil in conjugated fatty acids and oils [14]. 

Conjugated fatty acids and oils are indeed attractive substrates for various industrial uses [14] having, moreover, faster drying rates, better resistance to water, and improved toughness [17,18,19,20]. Conjugated oils are, for example, very valuable as coating components in paints, varnishes, and inks [21,22]. Related to organic reactivity, conjugated polyunsaturated fatty acids and esters can be more easily polymerized by thermal treatment under oxygen-free conditions: dimer, trimer and/or polymer fatty acids (or methyl esters) have been prepared by condensation of unsaturated monomers at high temperature (250 °C) in the presence or absence of suitable acid catalysts (clays, Lewis acids) [23,24].

**Scheme 1:**
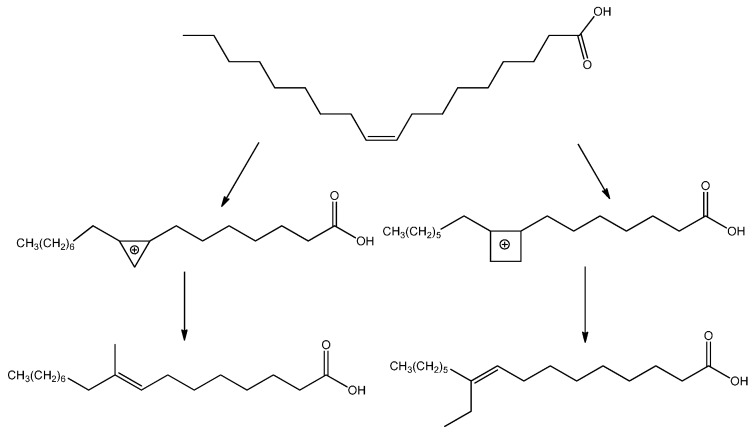
Proposed mechanism for fatty acid branching.

A number of reactions, including elaidinization, isomerization, chain branching, disproportionation, hydrogenation, and alkylation can, however, occur beside polymerization under thermal treatment of long-chain monosaturated fatty acids, in the presence of catalysts [23]. Branched monomeric fatty acids and esters are indeed normally produced as by-products of the dimer acid production process starting from unsaturated fatty acids. To improve yield and conversion in branched-chain isomers, zeolites [24,25] and mesoporous gallium-nibium mixed oxides [26] have been recently employed. However, acid-catalyzed reactions such as double bond and skeletal isomerization and dimerization, or redox processes such as dehydrogenation of methyl oleates to methyl linoleates, also occur over the mesoporous Ga-Nb oxides. The mechanism for the branching of unsaturated fatty acids, represented in Scheme 1, has been proposed [27] to proceed through the formation of a positively charged ring structure at the double bond followed by the ring breaking and reformation of the double bond with a methyl or ethyl side chain.

**Scheme 2:**
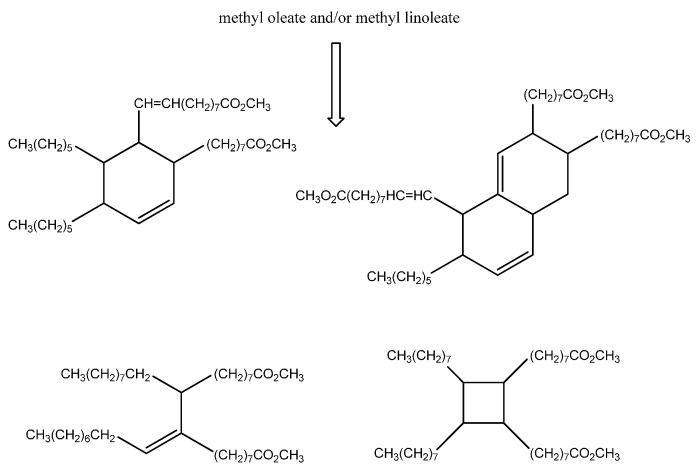
Cyclic and linear compounds arising from fatty acid ester dimerization.

Carbocations or radical intermediates are probably also involved in the dimerization reactions, giving either linear or cyclic compounds, although concerted Diels Alder mechanisms have been invoked more frequently to explain the formation of cyclohexene-based dimers and trimers (Scheme 2) [28]. The occurrence of the latter mechanism in thermal oil polymerization is, however, still a subject of debate [29].

Although most of the above-mentioned reactions are generally carried in molecular solvents, in the last 10 years, ionic liquids (ILs) have also been proposed as media and/or catalysts to transform fatty acids and related methyl esters in higher-value fine chemicals. Today, the term ILs defines a large class of compounds constituted exclusively by ions, liquid at/or near room temperature, that are characterized by a high thermal and chemical stability, a peculiar non-volatility, good solvent properties, and (depending on structure) catalytic abilities [30]. In the specific case of fatty acid transformation, Brønsted-Lewis acidic ILs, such as (3-sulfonic acid)propyltriethylammonium chlorozincates [HO_3_S-(CH_2_)_3_-NEt_3_]Cl-ZnCl_2_ (*x* = 0.64, *x*: molar fraction of ZnCl_2_), have been successfully used as solvents and catalysts for biodiesel dimerization [31]. Nonetheless, Lewis acidic ILs, constituted by a metal(III) chloride and an organic halide salt, have been employed for the preparation of branched fatty acids and esters starting from saturated [32] and unsaturated compounds through dimerization and polymerization processes [33]. Finally, methyl (ethyl) linoleate and soybean oil have been efficiently transformed into their conjugated isomers (linoleic derivatives) using transition metal complexes (RuHCl(CO)(PPh_3_)_3_ or RhCl(PPh_3_)_3_) in some common ILs, based on imidazolium, pyrrolidinium, and tetrabutylammonium cations associated with bromide, hexafluorophosphate, and bis(trifluoromethylsulfonyl)imide anions [34].

With the aim of producing useful branched chemicals from long-chain unsaturated esters, we decided to investigate the alkylation of methyl linoleate with propene using selected metal salts in ILs. In particular, we decided to investigate the catalytic ability of GaCl_3_ in chloride-based ILs and of metal bis(trifluoromethylsulfonyl)imide salts, M(Tf_2_N)_3_, in [bmim][Tf_2_N]. 

**Scheme 3:**
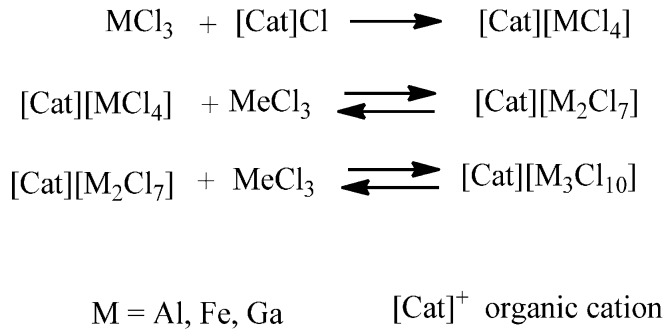
Equilibria in chlorometallate ILs.

The addition of a proper amount of a metal halide to a chloride- or bromide-based IL is indeed a simple and efficient way to obtain halometallate ILs able to act as solvents and Lewis acids. The reaction mixture may contain, depending on reagent concentration and temperature, beside a well-defined organic cation, halide and one or more ionic complexes (halometallates) in a dynamic equilibrium with each other [35]. These equilibria represent the main peculiarity of this important class of ILs, which have widely used as catalysts: anion speciation and, consequently, IL catalytic activity [36] strongly depends on metal features and concentration (Scheme 3) [37,38]. Chlorometallated ILs based on aluminum have been extensively investigated since the early 1980s, [39,40] but more recently other metal halides (indium(III), gallium(III) and iron(III), iron(II), copper(II), tin(II) and zinc(II)) have also been successfully used [41,42,43]. 

Chloroaluminate ILs are extremely moisture-sensitive and, in contact with traces of water, hydrolyze releasing hydrogen chloride. Consequently, the catalytic activity of chlorogallate(III) ILs, less sensitive to hydrolysis, was tested in the present investigation. 

Furthermore, since a reactive dissolution of metal salts can also occur in ILs constituted by fewer coordinating anions, such as [Tf_2_N]^−^, the catalytic ability of some metal bis(trifluoromethylsulfonyl)imides in [bmim][Tf_2_N] was investigated. Metal salts in these media result in negatively charged metal-containing species, [M(Tf_2_N)_x+1_]^−^ [44], for which the ability to act as catalysts and media in some Lewis acid-catalyzed processes has been shown [45].

The alkylation of fatty acids by co-polymerization with alkenes has been scarcely investigated in molecular solvents as well. To the best of our knowledge, there is only one example in the literature describing the synthesis of internal branched acids by the addition of ethylene to linoleic acid or the corresponding conjugated 18C:2 fatty acids using rhodium as a catalyst and hexane as a solvent [46,47].

Here, we show that the catalytic properties of some ILs can provide new opportunities to synthetize valuable branched high-molecular-weight products by addition of a terminal alkene (propene) to unsaturated fatty acid methyl esters or by dimerization-oligomerization processes.

## 2. Results and Discussion

**Scheme 4:**
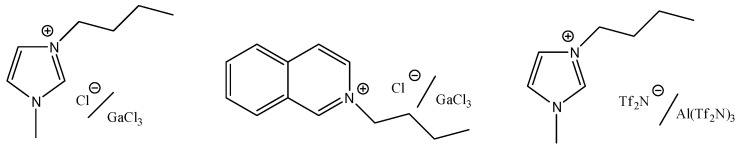
Lewis acidic IL mixtures.

Preliminarily, to evaluate the possibility of obtaining branched fatty acid methyl esters by alkylation of methyl linoleate with propene, we have investigated the catalytic activity of three metal salt-IL systems. In particular, we have tested the Lewis acidic mixtures arising from the addition of a proper amount of GaCl_3_ (Χ > 0.5) to two chloride-based ILs (1-butyl-3-methylimidazolium chloride, [bmim]Cl, and 1-butylisoquinolium chloride, [BuIsoq]Cl) or by dissolution of a smaller amount of Al(Tf_2_N)_3_ (Χ = 0.1) in 1-butyl-3-methylimidazolium bis(trifluoromethylsulfonyl)imide, [bmim][Tf_2_N] (Scheme 4).

Chlorogallate ILs, although more stable to hydrolysis than the corresponding aluminum salts, remain moisture-sensitive. Nonetheless, metal bis(trifluoromethylsulfonyl)imides in [Tf_2_N]-based ILs give moisture-stable systems, which are, however, highly hygroscopic. Therefore, to avoid (or reduce) unwanted effects attributable to the presence of water, all the metal salt-ILs mixtures have been prepared starting from rigorously dried ILs and metal salts, in a glovebox under argon atmosphere. 

Reactions of methyl linoleate with propene have been carried out in a four-position organic synthesizer process station, under controlled temperature and pressure (Table 1). Products have been always extracted with hexane, although in [BuIsoq]Cl the reaction products form a separate phase from the ionic liquid. The recovered products, whose weights were generally comparable or slightly higher than that of the starting fatty acid ester, always resulted largely free of any catalyst or ionic liquid contamination. 

**Table 1:** Reactions of methyl linoleate with propene under pressure at 100 °C.

Run	ILs	MX_3_	Χ ^a^	Propene Pressure (bar)	Time (h)
1	[bmim][Tf_2_N]	Al(Tf_2_N)_3_	0.1	7	24
2	[bmim][Tf_2_N]	Al(Tf_2_N)_3_	0.1	7	72
3	[bmim]Cl	GaCl_3_	0.6	7	8
4	[BuIsoq]Cl	GaCl_3_	0.6	7	8

^a^ Χ = n_(metal salt)_/[n_(metal salt)_ + n_IL_].

**Figure 1:**
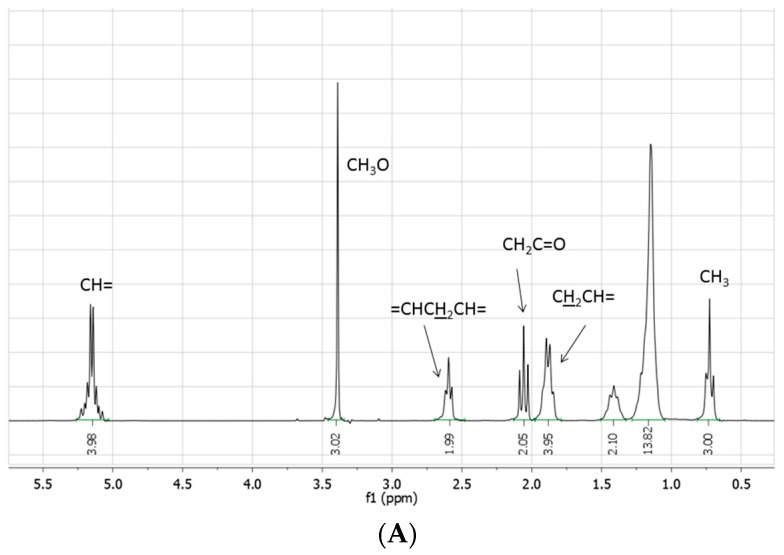

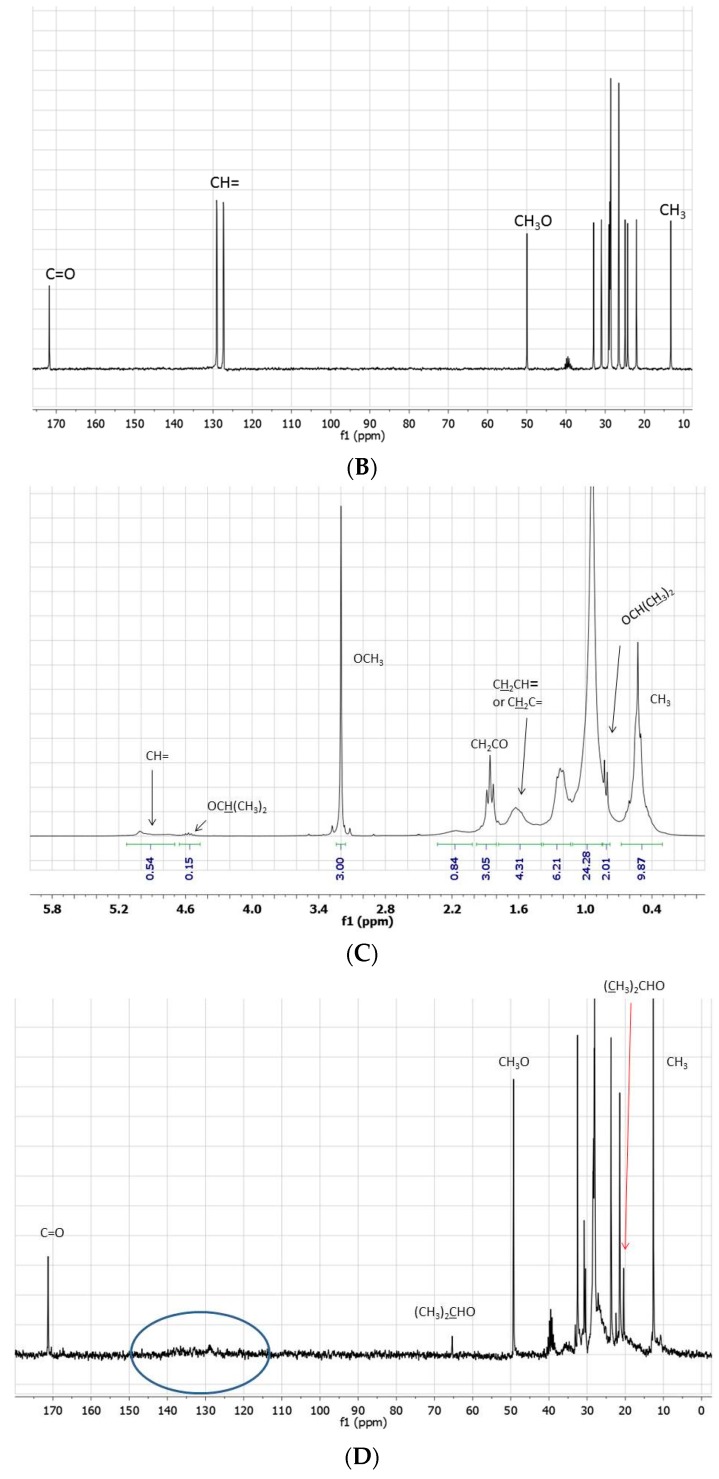

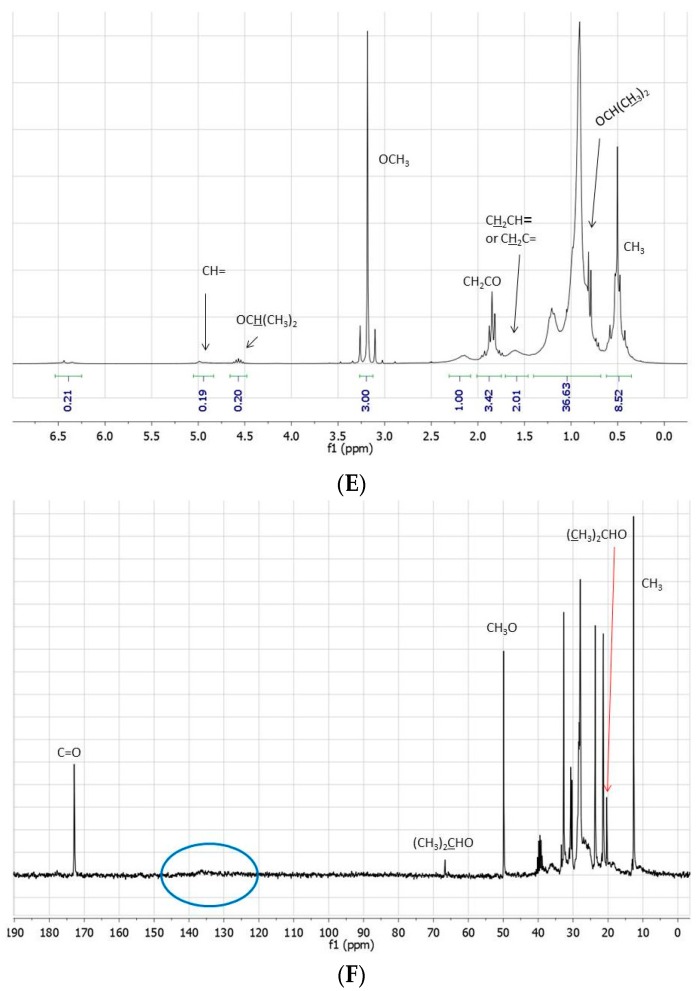
(**A**) ^1^H- and (**B**) ^13^C**-**NMR spectra (neat, DMSO-*d*_6_ in coaxial tube) of methyl linoleate; (**C**) ^1^H- and (**D**) ^13^C**-**NMR spectra (neat, DMSO-*d*_6_ in coaxial tube) of the product mixture arising from the reaction of methyl linoleate with propene in [bmim][Tf_2_N]/Al(Tf_2_N)_3_ at 100 °C for 72 h; (**E**) ^1^H- and (**F**) ^13^C-NMR spectra (neat, DMSO-*d*_6_ in coaxial tube) of the product mixture arising from the reaction of methyl linoleate with propene in at 100 °C [bmim][GaCl_4_/Ga_2_Cl_7_ for 8 h. The blue circles evidence the region of the double bond carbons.

The crude reaction mixtures were analyzed by TLC, IR, NMR, GC-MS, and ESI-MS. With the exception of the reaction reported in entry 1, for which IR, TLC, and NMR analyses evidence only a moderate transformation of the starting fatty acid ester, methyl linoleate was not detected in the other reactions: the NMR spectra indeed showed the disappearance of the bisallylic hydrogen signal as well as of the related carbon atom. The same spectra were moreover characterized by: (i) the presence of small (if any) signals around δ 5.0 ppm attributable to vinylic protons, whose integral was significantly reduced with respect to the starting methyl linoleate; (ii) the presence of signals between 1.6–2.2 ppm attributable to several allylic methylene (or methyne) protons; (iii) a signal at 0.5 ppm attributable to terminal methyl groups, significantly increased with respect to the starting methyl linoleate; (iv) the presence of a multitude of small signals due to aliphatic carbons between 20 and 35 ppm; (v) the presence of hydrogen signals at 4.6 and 0.8 ppm attributable to a (CH_3_)_2_CHO- group, confirmed by the presence signals at 65.8 and 20.8 ppm in the ^13^C-NMR spectrum (Figure 1).

All these features, associated with an increase of the signals due to aliphatic protons (including the terminal CH_3_), exclude the sole occurrence of simple double bond isomerization processes, or the exclusive formation of methyl linoleate dimers and trimers, as well as the sole involvement of Diels Alder cycloadditions.

**Figure 2:**
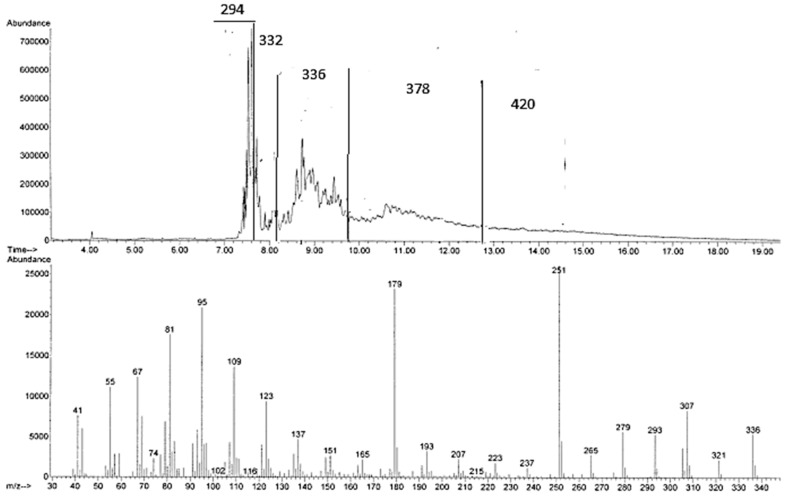
GC-MS chromatogram and MS spectrum of one of the signals with M^+^ 336 arising from the reaction of methyl linoleate with propene in [bmim][Tf_2_N]/Al(Tf_2_N)_3_ at 100 °C for 72 h.

On the other hand, the direct GC-MS analysis of the crude products arising from the reactions in [bmim][Tf_2_N]/Al(Tf_2_N)_3_ (Figure 2) and [bmim][GaCl_4_/Ga_2_Cl_7_ suggests the presence of mixtures of fatty acid methyl ester isomers with M^+^ = 294 *m*/*z*, 336 *m*/*z* (corresponding to methyl linoleate + propene), M^+^ = 378 *m*/*z* (corresponding to methyl linoleate + 2 propene), and M^+^ = 420 *m*/*z* (corresponding to methyl linoleate + 3 propene). Actually, the presence of peaks with M^+^ = 294 *m*/*z*, corresponding to methyl linoleate or its isomers, is surprising. It is indeed in disagreement with the NMR spectra, which show the absence of methyl linoleate and its isomers! The possibility that these peaks might be due to the decomposition of higher-molecular-weight compounds is, however, a sound hypothesis corroborated by the chromatogram shape showing typical degradation patterns.

**Figure 3:**
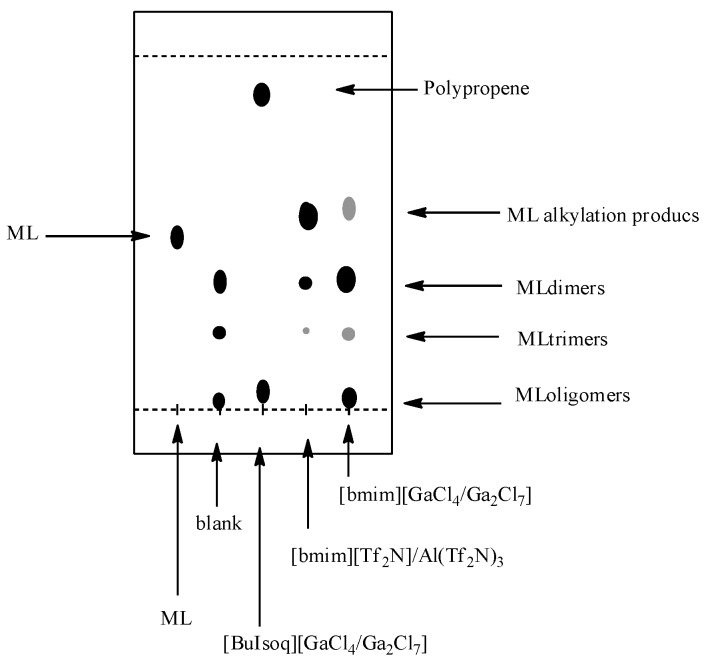
TLC analysis of the reactions between methyl linoleate and propene in [BuIsoq][GaCl_4_/Ga_2_Cl_7_], [bmim][Tf_2_N]/Al(Tf_2_N)_3,_ and [bmim][GaCl_4_/Ga_2_Cl_7_]. ML: pure methyl linoleate; Blank: experiment carried out in [bmim][GaCl_4_/Ga_2_Cl_7_] without propene.

However, relevant information can also be obtained by TLC analysis (Figure 3). TLC plates (Silicagel 60 on Al foil; eluent hexane–ethyl acetate, 9:1) of the reactions indeed present two or three main spots: the first, with Rf slightly higher than that of methyl linoleate and more evident for the reaction in [bmim][Tf_2_N]/Al(Tf_2_N)_3_; the second and the third, with R_f_ significantly lower than that of methyl linoleate and more evident in [bmim][GaCl_4_/Ga_2_Cl_7_], corresponding to those observed in the blank experiment always carried out in [bmim][GaCl_4_/Ga_2_Cl_7_], but in the absence of propene.

For this latter reaction, the crude products (quantitatively recovered and corresponding to the two spots at lower R_f_ in TLC analysis) have been identified on the basis of the ESI-MS spectra, as dimeric (DimerH^+^ + H_2_O = 609 *m*/*z*) and trimeric adducts (TrimerH^+^ + H_2_O = 903 *m*/*z*) of the starting methyl ester, containing two and three double bonds, respectively. The ability of GaCl_3_ to also give hydrogen transfer reactions has been recently proven [48]. 

**Figure 4:**
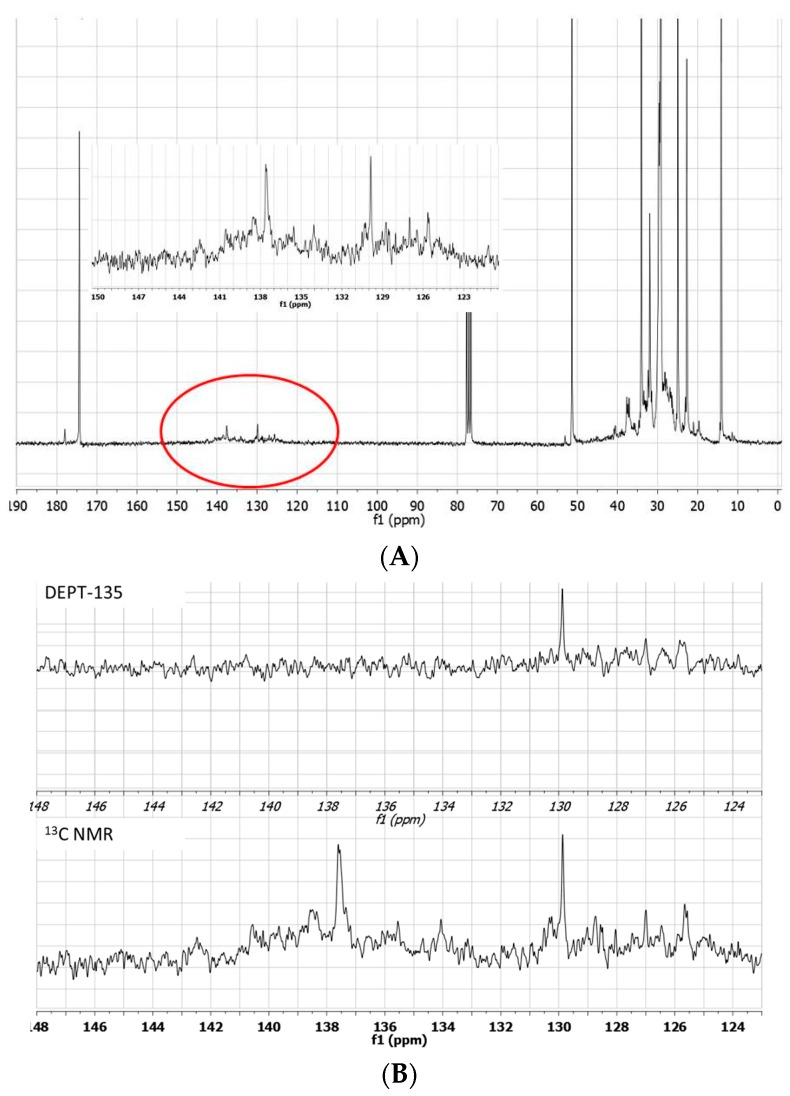
^13^C-NMR spectrum of the blank experiment carried out in [bmim][GaCl_4_/Ga_2_Cl_7_] in the absence of propene. Insert shows the region circled in the spectrum (**A**). ^13^C-NMR and DEPT-135 expanded spectra of the region between 148–123 ppm (**B**).

It is noteworthy that the ^1^H-NMR spectra of this reaction mixture confirm that most of the olefinic protons are lost: the integral of these protons, also including signals shifted between 6.5–7.0 ppm, comes out significantly decreased with respect to that of the starting material (Figure 4A). Moreover, the comparison ^13^C-NMR/DEPT spectra show the presence of several quaternary olefinic carbons in the region between 125–140 ppm (Figure 4B).

**Figure 5:**
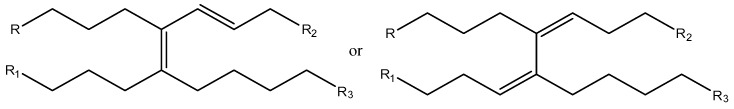
Isomeric products containing tri and or tetrasubstituted double bonds.

These data suggest that methyl linoleate oligomerization occurs in [bmim][GaCl_4_/Ga_2_Cl_7_] with the formation of isomeric products containing tri and or tetrasubstituted double bonds (Figure 5).

Unfortunately, methyl linoleate dimers, trimers, and higher oligomers cannot be detected by GC-MS. However, depending on column conditions, it is possible to show, in the region corresponding to methyl linoleate and methyl oleate, a number of peaks with an apparent M^+^ 297, probably due to degradation products. Such decomposition probably also occurs when the reaction of methyl linoleate and propene is analyzed under analogous conditions, thus explaining the above-mentioned conflicting results between NMR and GC-MS.

It is noteworthy that NMR spectra and the GC-MS chromatogram clearly indicate the presence of transesterification products, *i.e.*, isopropyl esters (lower than 10%), in particular in [bmim]Tf_2_N/Al(Tf_2_N)_3_. The formation of these products can be rationalized assuming the *in situ* generation of isopropanol from propene and traces of water, despite the fact that dry reagents and solvents were employed under anhydrous conditions. Since, surprisingly, the presence of a residual humidity appeared unable to significantly affect the catalytic activity of the investigated systems, the already accurate drying process was not further improved.

Finally, a detailed discussion is required for the reaction in [BuIsoq][GaCl_4_/Ga_2_Cl_7_]. In this medium, at variance with [bmim][Tf_2_N]/Al(Tf_2_N)_3_ and [bmim][GaCl_4_/Ga_2_Cl_7_], the formation of polypropene and methyl linoleate oligomers is the dominating process. Despite the fact that the two gallium-based ILs contain the same molar amount of GaCl_3_, these present a significantly different catalytic ability: [BuIsoq][GaCl_4_/Ga_2_Cl_7_] is extremely more efficient than [bmim][GaCl_4_/Ga_2_Cl_7_] in propene oligomerization. 

On the basis of these data, [bmim][Tf_2_N]/Al(Tf_2_N)_3_ appears the most suitable medium in which methyl linoleate alkylation with propene can compete with methyl linoleate or propene oligomerization.

In order to quantify the linoleate alkylation products formed in [bmim][Tf_2_N]/Al(Tf_2_N)_3_, the crude reaction mixture was separated into six fractions by column chromatography on silica gel. The fractions were analyzed by GC-MS and NMR. The first three fractions (*ca.* 40%–45% of the crude mixture) essentially contain the expected products arising from the addition of one or two propene molecules to methyl linoleate or to its corresponding isopropyl ester. Actually, these fractions, when analyzed by GC-MS, give a series of broad peaks (some probably due to decomposition, M^+^ 294), which have been attributed to products (with M^+^ 336, 378, and 420 *m*/*z*) arising from the addition of one or more propene molecules to the *in situ* formed methyl linoleate isomers (see above). Linoleic acid consists of 18 carbon atoms, with two cis double bonds located on carbon 9 and 12: in the presence of suitable catalysts (Lewis and Brønsted acids) double bond conjugation is associated with the formation of a mixture of positional and geometric isomers. In principle, 56 different conjugated linoleic acid isomers are possible, *i.e.*, 14 positional isomers with conjugated double bonds located on carbons from 2 and 4 to carbons 15 and 17, each of them with the conjugated double bonds in *cis*/*cis*, *cis*/*trans*, *trans*/*cis*, or trans/trans configuration. Reasonably, under the employed reaction conditions, the first step of the investigated process is the rearrangement of methyl linoleate into the corresponding isomers possessing more reactive conjugated double bonds.

On the other hand, the subsequent formation of a large number of addition products, from the *in situ* formed conjugated isomeric linoleate methyl esters, can account for the dispersion of the vinylic signals in the NMR spectra and, therefore, for the fact that vinylic protons and carbons are not evident in these spectra. The ^1^H-NMR spectra of these fractions are, however, characterized by a significant increase in intensity of the signals related to the aliphatic protons (particularly evident that of terminal CH_3_-) with respect to the CH_3_O- signal, whereas new signals between 25 and 40 ppm can be observed in the ^13^C-NMR spectra. 

Consequently, GC-MS and NMR spectra strongly support the formation of the expected alkylation products: branching position and double bond position and geometry, however, cannot be established due to the high number of possible isomers. 

**Figure 6:**
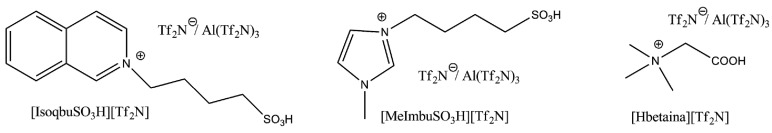
Brønsted/Lewis acidic ILs mixtures.

Since Brønsted acidic ILs consisting of imidazolium cations with alkane sulfonic acid side chains have been found to be active catalysts in some dimerization processes (for example, that of α-methylstyrene) [49], we decided to test also the activity of Al(Tf_2_N)_3_ in other three properly functionalized Brønsted acidic ILs (Figure 6). 

The reactions, carried out in [IsobuSO_3_H][Tf_2_N]/Al(Tf_2_N)_3_ [MeImbuSO_3_H][Tf_2_N]/Al(Tf_2_N)_3_ and [Hbetaine][Tf_2_N]/Al(Tf_2_N)_3_ with propene, under pressure (7 bar) at 100 °C for 24 h, gave product mixtures qualitatively similar to those isolated by the reaction in [bmim][Tf_2_N]/Al(Tf2N)_3_, although characterized by the formation of more significant amounts of isopropyl esters.

Also, some attempts to increase the yields in alkylation products changing the metal salt failed: practically, unreacted methyl linoleate was isolated from the reactions carried out in [bmim][Tf_2_N] using Ni(Tf_2_N)_2_ and Y(Tf_2_N)_3_ as a catalyst whereas dimerization, alkylation, and significant transesterification occurred in the presence of Cu(Tf_2_N)_2_. 

Finally, we must mention that we have reused the catalytic system [bmim][Tf_2_N]/Al(Tf_2_N)_3_ three times, after drying at 80 °C under vacuum for 24 h, without any significant change in the catalytic activity or selectivity. Conversion and selectivity (*i.e.*, methyl linoleate alkylation/oligomerization) were almost constant throughout the runs (checked by NMR and GC-MS). This indicates that the characteristics of the catalytic species in the ionic liquid phase is not affected by the removal of the organic phase.

In summary, during this investigation aiming to explore the possibility of adding propene to methyl linoleate, we have surprisingly found that in [bmim][GaCl_4_/Ga_2_Cl_7_] the reaction occurs prevalently with methyl linoleate oligomerization affording branched long-chain diesters and triesters. At variance, in [bmim][Tf_2_N]/Al(Tf_2_N)_3_, alkylation with propene can compete significantly with methyl linoleate oligomerization. On the other hand, in [BuIsoq][GaCl_4_/Ga_2_Cl_7_], propene oligomerization appears as the dominating process beside linoleate oligomerization. Despite the anhydrous conditions employed in this work, isopropyl esters have been found as by-products in these reactions. Moreover, their formation significantly increases when Brønsted-Lewis acidic ILs ([IsobuSO_3_H][Tf_2_N]/Al(Tf_2_N)_3_ [MeImbuSO_3_H][Tf_2_N]/Al(Tf_2_N)_3_ and [Hbetaine][Tf_2_N]/Al(Tf_2_N)_3_) have been used.

## 3. Materials and Methods 

NMR spectra were recorded at room temperature using a Bruker instrument at 250 MHz (^1^H) and 75.7 MHz (^13^C), in DMSO-*d*_6_, CDCl_3_, or D_2_O. The progress of the reactions was followed by comparison of the areas of the bisallylic hydrogen triplet (2.7 ppm) and the alpha carbonyl CH_2_ triplet (2.30 ppm) or the OCH_3_ singlet signal. The allylic region at 1.90–2.4 ppm was used as proof of the formation of conjugated isomers. Transesterification was estimated by comparing the (CH_3_)_2_CH-O methylenic hydrogen in isopropyl esters and the OCH_3_ protons of the corresponding esters. 

ESI-MS spectra were recorded on a Finnigan LCQ Advantage (Thermo Finnigan, San Jose, CA, USA) ion trap instrument equipped with Excalibur software. Solid samples were dissolved in proper volume of dichloromethane (HPLC grade) and injected directly by a pump syringe (5 μL/min) in the ESI-MS system. GC-MS analyses were performed with a Varian CP-3800 gas-chromatograph (Varian, Inc., Walnut Creek, CA, USA) equipped with a DB-5 capillary column (30 m × 0.25 mm; coating thickness 0.25 µm, (Agilent Technologies, Santa Clara, CA, USA) and a Varian Saturn 2000 ion trap mass detector Varian, Inc., Walnut Creek, CA, USA. Analytical conditions: injector and transfer line temperatures at 250 and 240 °C, respectively; oven temperature was programmed from 60 °C to 240 °C at 3 °C/min; carrier gas helium at 1 mL/min. Infrared were registered using a FTIR Agilent 660 (Agilent Technologies, Santa Clara, CA, USA). The compounds 1-Butyl-3-methylimidazolium chloride ([bmim]Cl), 1-butyl-3-methylimidazolium bis(trifluoromethylsulfonyl)imide ([bmim][Tf_2_N] and 1-butylisoquinolium chloride ([BuIsoq]Cl) were synthesized as previously reported [50]. The [Betaine][Tf_2_N] was prepared by addition of an equimolar amount of HTf_2_N to betaine (2 g, 98%) in water. The [IsobuSO_3_H][Tf_2_N] and [MeImbuSO_3_H][Tf_2_N] were synthesized by reaction of the corresponding amines (isoquinoline and methylimidazole, respectively) with an equimolar amount of 1,4-butane sultone (>99%) at room temperature in the absence of solvent, followed by addition of an equimolar amount of HTf_2_N. Methyl linoleate (98%) and GaCl_3_ (>99% pure) were used as supplied, Al(Tf_2_N)_3_ was dried before use under vacuum for 8 h at 80 °C.

All reactions and manipulations were carried out under a dry argon atmosphere using standard Schlenk, vacuum-line, and glovebox techniques. Reactions were carried out in the all-steel Eyela four-position organic synthesizer process station able to perform pressurized reactions under magnetic stirring and temperature control.

### Alkylation Procedure

In a glovebox, under argon flow, the proper metal salt (1.1 mmol of M (Tf_2_N); 15 mmol of GaCl_3_) was added to the glass reaction vessel containing the IL (*ca.* 4 g, 10 mmol), previously dried at 80 °C under vacuum (6 h, 0.001 mmHg). The resulting mixture was magnetically stirred, under argon atmosphere, until a homogeneous system was obtained. Thus, methyl linoleate (3 mL, 11 mmol) was added and the vessel was inserted in the steel apparatus and firmly closed. After the introduction of propene (7–9 bar), and/or eventually argon (6–7 bar), the mixture was slowly warmed and maintained with 500−600 rpm stirring at the reaction temperature (100 °C) for 5–24 h. After the desired reaction time, the apparatus was cooled and opened. The organic layer was decanted off or extracted with *n*-hexane. After solvent removal at reduced pressure, the crude oil was analyzed by IR, NMR and GC-MS. 

Products were purified by column chromatography on silica gel using as eluent mixtures of hexane-ethyl acetate (from 2% to 10%).
